# Patterns of cognitive decline and somatosensory processing in a mouse model of amyloid accumulation

**DOI:** 10.1016/j.ynpai.2021.100076

**Published:** 2021-11-07

**Authors:** Olivia Uddin, Keiko Arakawa, Charles Raver, Brendon Garagusi, Asaf Keller

**Affiliations:** Department of Anatomy and Neurobiology, Program in Neuroscience, University of Maryland School of Medicine, 20 Penn Street, Baltimore, MD 21201, United States

**Keywords:** Pain, Formalin Pain, Amyloid, 5XFAD

## Abstract

Pain and cognitive decline increase with age. In particular, there is a troubling relationship between dementia and pain, with some studies showing higher prevalence and inadequate treatment of pain in this population. Alzheimer’s disease (AD) is one of the most common causes of dementia in older adults. Amyloid plaques are a hallmark of AD. The downstream processes these plaques promote are believed to affect neuronal and glial health and activity. There is a need to better understand how the neuropathological changes of AD shape neural activity and pain sensitivity. Here, we use the 5XFAD mouse model, in which dense amyloid accumulations occur at early ages, and in which previous studies reported signs of cognitive decline. We hypothesized that 5XFAD mice develop sensory and pain processing dysfunctions. Although amyloid burden was high throughout the brain, including in regions involved with sensory processing, we identified no functionally significant differences in reflexive or spontaneous signs of pain. Furthermore, expected signs of cognitive decline were modest; a finding consistent with variable results in the literature. These data suggest that models recapitulating other pathological features of Alzheimer’s disease might be better suited to studying differences in pain perception in this disease.

## Highlights

• Despite copious amyloid plaques, 5XFAD mice show modest signs of cognitive decline.
• At ages 2 to 13 months old 5XFAD mice show no signs of sensory or pain dysfunctions.
• 5XFAD mice may not be a valid model for pain abnormalities in the context of AD.

## Introduction

### Pain in dementia is a growing concern

As the US population ages, aging-related health changes urgently require greater understanding and rigorous investigation. Older individuals encounter an increased risk of both pain and dementia, with both conditions having heavy economic and social burdens (Tsang et al., 2008, Duncan et al., 2011). Older adults with dementia experience particularly high levels of pain prevalence (Li et al., 2015, van Kooten et al., 2015) and some research identifies correlations between pain prevalence and cognitive decline (Whitlock et al., 2017). Especially concerning, pain is often undiagnosed and under-treated in patients with dementia, further complicating the interaction between cognitive function and pain (Li et al., 2015, Hadjistavropoulos et al., 2014). There is compelling but incomplete evidence that pain prevalence might differ in patients with varying dementia etiologies (reviewed by Binnekade et al., 2017). Thus, an aging population requires us to better understand how pain processing and cognitive decline change in distinct models of dementia, compared to normal aging.

### Pain in Alzheimer’s Disease

Alzheimer's disease is the leading cause of dementia in the aged. Some studies have found heightened functional connectivity between affective pain-processing brain regions and lowered pain tolerance in individuals with Alzheimer's disease (Cole et al., 2006, Colel et al., 2011), while others have found higher pain tolerance in patients with Alzheimer's (Benedetti et al., 1999). One method of disentangling the pain processing trajectory in Alzheimer’s disease is to investigate how neuropathological hallmarks like amyloid plaque accumulation (Gale et al., 2018, Garre-Olmo, 2018) affect pain sensitivity. Amyloid plaque burden is compelling for further study because it may shape neuronal activity, which in turn can alter sensory and pain processing. In both humans (Zott et al., 2018) and animals, amyloid proximity correlates with neuronal hyperactivity (Busche, 2012, Gurevicius et al., 2013, Busche and Konnerth, 2016, Zott et al., 2019). Downstream consequences of amyloid accumulation, such as glial activation (Barger and Harmon, 1997, Pasqualetti et al., 2015, Hansen et al., 2018) or neuronal death (Klyubin, 2008, Mattson, 1997, Smale et al., 1995, Kobayashi et al., 2002, Kobayashi et al., 2005), might also shape pain perception.

### 5XFAD animals model amyloid accumulation.

Here, we use the 5XFAD mouse model (Oakley, 2006) of early amyloid accumulation to evaluate early-onset changes in sensory function and pain processing. 5XFAD animals carry five familial Alzheimer’s disease mutations in genes coding for amyloid production and processing proteins (presenilin-1 and amyloid precursor protein). By an early age (2 months) they develop glial activation and striking deposits of both extracellular amyloid plaques and intraneuronal amyloid (Oakley, 2006). While there are reports of modest cognitive decline in 5XFAD animals, the severity and onset of these changes are widely variable among studies. There are no reports known to us that thoroughly characterize sensory processing or pain sensitivity in 5XFAD animals. We find that, in 5XFAD mice, signs of cognitive decline are modest or absent up to 13 months of age, and that these animals show no distinct profile of altered pain sensitivity.

## Methods

### Rigor.

We adhered to accepted standards for rigorous study design and reporting to maximize the reproducibility and translational potential of our findings as described by Landis et al. (2012) and in ARRIVE (Animal Research: Reporting In Vivo Experiments).

### Animals

All procedures were performed in accordance with the Animal Welfare Act regulations and Public Health Service guidelines and approved by the University of Maryland School of Medicine Animal Care and Use Committee. 5XFAD mice (B6SJL-Tg(APPSwFlLon,PSEN1*M146L*L286V)6799Vas/Mmjax) were purchased from the Jackson Laboratory (Bar Harbor, ME) and maintained by crossing with B6SJLF1/J (Jackson Laboratory) in our animal facility. Offspring of 5XFAD were housed up to 5 animals with same sex littermates including heterozygous transgenic (Tg) and wild type (WT) animals. To determine their genotype, tail clip samples were sent to TransnetYX (Cordova TN) where they were processed through PCR with APPsw gene and huPSEN1 gene probes. We studied 139 animals in total (72 controls and 67 5XFAD). Control animals were wild-type littermates of 5XFAD mice.

### Y-maze

**Fig. 1:**
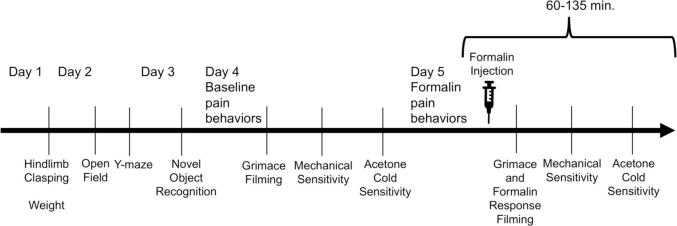
Timeline of behavioral testing. Hindlimb clasping scores and weights were logged on day 1. Cognitive behaviors were tested on days 2 and 3, with pain-related behaviors following on days 4 and 5. On days when pain sensitivity was tested, grimace filming was performed first, followed by mechanical sensitivity testing, then acetone cold testing.

To assess spatial memory, we used the Y-maze behavioral test, as described previously (Mast et al., 2017). Animals were acclimated to the experimental room for at least 20 min; they did not undergo habituation or training in the maze prior to testing, because the test relies on analyzing exploration of a novel environment (Ohno et al., 2004). Each animal was then placed into the center of the plexiglass maze (arm widths of 5 cm, lengths of 35 cm, and heights of 10 cm). While filming, we observed animals in the maze and noted which arm they entered (to be counted as an entry, all four paws had to be within the limits of the maze arm). Animals were permitted to move freely for at least 8 min, or until the number of maze arm entries reached 20. We then calculated the percent of alternation by taking the number of entries into another arm from the previous arms divided by the total number of entries into all arms. Fig. 1 outlines the order of all behavioral testing across days.

### Novel object recognition

We used the novel object recognition test to assess short term memory. We allowed animals to acclimate to the experimental room for at least 20 min. One at a time, we placed the animals into a clean home cage (15 cm × 30 cm × 15 cm) with a thin layer of corn-cob bedding for a 3-minute habituation period. We then introduced two identical objects into the cage for 5 min. One hour later, we re-introduced animals into the cage for 3 min, this time with one familiar object and one novel object of similar size. We manually assessed videos, quantifying the number of seconds each animal spent exploring the new object (defined as directed sniffing or physical contact) compared to the familiar object. We then calculated the time spent exploring the new object as a ratio of the total time spent exploring.

### Open field

As described previously (O'Leary et al., 2020, Braun and Feinstein, 2019), we used open field to quantify general locomotion and anxiety-like behavior in mice in a novel environment. We allowed animals to acclimate to the experimental room for at least 20 min; they did not undergo a habituation period in the open field box and each animal was tested only once. While filming from above, we placed each animal into the center of the open field (50 cm long square apparatus with 38 cm high walls). Animals were allowed to move freely for 10 min. To calculate distance traveled, velocity, time spent in the inner region of 30 cm × 30 cm, time spent in the outer region, and time spent immobile (>2 s of non-locomotion) we used the AnyMaze software (Stoelting Co., Wood Dale, IL).

### Hindlimb clasping

We used the hindlimb clasping test to assess sensorimotor function in aging mice, as previously described (Guyenet et al., 2010). Holding animals by the tail, we lifted them for 10 s and scored them based on the following criteria: 0 for splayed hindlimbs with no clasping, 1 for one hindlimb being retracted for at least half of the observation time, 2 for both hindlimbs being partially retracted for more than half of the observation time, and 3 for both limbs being entirely retracted, touching the abdomen, for more than half of the observation time.

### Formalin injection and response scoring

To induce persistent inflammatory pain, we subcutaneously injected 10 µl of 5% Formalin (Sigma Millipore, Burlington, MA) into the dorsum of the right hindpaw. We then filmed animals for 30 min to assess formalin-evoked behaviors (Abbott et al., 1995) and to simultaneously obtain face screenshots for grimace scoring. To determine the phase one and phase two responses, we divided the 30-minute video into the first 10 min (phase 1) and the final 20 min (phase 2). To quantify the number of formalin-evoked behaviors per minute, we scored each segment of 15 s. If any guarding for more than 2 s, paw licking, or paw flicking occurred, the animal was given one point. We then calculated the number of behaviors per minute based on these scores and the total time of filming. For a given 15-second segment, the maximum score possible was one point, such that the highest possible score per minute for any given animal was 4 behaviors per minute.

### Grimace scoring

To assess ongoing, spontaneous pain, we used the grimace scoring system (Langford et al., 2010, Sotocinal et al., 2011, Akintola et al., 2017). On baseline and formalin behavior days, we filmed mice in plexiglass chambers (7 cm × 12 cm × 6.4 cm) for 30 min (immediately following formalin injection on days when formalin was administered). We obtained 10 images per animal by capturing video screenshots. We scored each action unit on a scale from 0 (minimal pain) to 2 (multiple signs of pain) for each image as previously described (Langford et al., 2010, Sotocinal et al., 2011). We used a custom-written MATLAB script (Natick, MA) which allowed all images from all timepoints to be scrambled so that the scorer was blind to timepoint and genotype. This MATLAB script then averaged each image’s score to calculate a “grand average” grimace score for each animal at each timepoint.

### Mechanical sensitivity

To assess mechanical sensitivity, we applied calibrated von Frey filaments (North Coast Medical, Gilray, CA) to the plantar surface of the right hindpaw. We defined a response as a sharp paw withdrawal, licking, or shaking, and used the up-down method across 10 trials (Dixon, 1965) to determine each animal’s withdrawal threshold. Trials where the response pattern did not converge to a stable value were discarded. To facilitate comparison between data collected by different experimenters, we converted response thresholds to Z-scores by subtracting the group mean from each individual threshold, divided by the group standard deviation. On formalin-administration testing days, mechanical sensitivity testing followed the 30-minute grimace filming session.

### Thermal sensitivity (Cold)

To assess cold sensitivity, we used a 1 mL syringe to apply 1 drop of acetone (Fisher Scientific, Hampton, NH) to the plantar surface of the hindpaw. We performed 3 trials of acetone application at least 5 min apart and scored as described previously (Flatters and Bennett, 2004, Park et al., 2018). Scores were then assigned, the minimum score being 0 (no response to any of the 3 trials) and the maximum possible score being 3 (repeated flicking and licking of paws on each of the 3 trials). We averaged the score for each trial to yield the total score for each animal. On formalin administration testing days, acetone testing followed mechanical sensitivity testing.

### Immunohistochemistry

Animals were perfused transcardially with 0.05 M PBS followed by 4% paraformaldehyde (Sigma Millipore). After harvesting brains, they were cryoprotected with 15% sucrose in PBS for 24 h, then in 30% sucrose for 24 h. Brains were frozen in OCT (Sakura Finetek, Torrance, CA) and sliced on a cryostat at 50 µm thickness (CM1860, Leica Biosystems, Buffalo Grove, IL). Sections were washed 5 times in 0.05 M PBS, then blocked with 4% normal donkey serum and 0.1% Triton X-100 in 0.05 M PBS for 1 h at room temperature. Then, sections were incubated in Rabbit anti amyloid-beta primary antibody at 1:3000 (Abcam ab2539, Cambridge, UK) for 48 h at 4 °C. Sections were then rinsed 5 times in 0.05 M PBS before being incubated in secondary antibody, Cy3 donkey anti rabbit, at 1:300 for 2 h at room temperature (Invitrogen/Thermofisher A-21121). Sections were washed 5 more times in 0.05 M PBS, incubated with DAPI in PBS, followed by one wash of PBS in the dark. Finally, sections were mounted on slides with a fluorescent mounting medium produced in-house. Images were acquired using a confocal microscope (SP8, Leica Biosystems, Buffalo Grove, IL).

### Statistical analysis

We analyzed all data and generated figures using GraphPad PRISM version 8.2.0 for Mac (GraphPad Software, La Jolla, CA). We used a 2-way ANOVA (or a Kruskal-Wallis test if parametric assumptions were not met) to analyze all data, with sex and genotype and the variables. Different animals comprised each age group, thus the experimental design was not a repeated measures design. Therefore, we analyzed each age group separately. In instances where there is a main effect or an interaction effect, we report partial η^2^ effect sizes with values ≥ 0.25 being considered large effect sizes, values ≥ 0.09 being medium and values ≥ 0.01 being small. For all tests, n represents the number of animals. All statistical data are included in the statistical table (Table 1).

**Table 1:** Statistical table. For each figure, the statistical test used, number of animals in each group (n), medians, and 95% confidence intervals.

Figure (metric)	Data Structure	Statistical Test	n; Medians (95% Confidence Intervals)
3 (body weight - grams)	Normal	2-way ANOVA	2 month male control: 8; 23.5 (22.0–24.0) 2 month male 5XFAD: 5; 24.0 (21.0–25.0) 2 month female control: 8; 17.5 (15.0–18.0) 2 month female 5XFAD: 8; 16.0 (15.0–18.0) 4 month male control: 7; 28.0 (26.0–36.0) 4 month male 5XFAD: 10; 29.5 (27.0–31.0) 4 month female control: 8; 19.0 (17.0–22.0) 4 month female 5XFAD: 8; 20.0 (16.0–22.0) 8 month male control: 9; 32.0 (29.0–40.0) 8 month male 5XFAD: 7; 33.0 g (25.0–43.0) 8 month female control: 9; 26.0 g (22.0–33.0) 8 month female 5XFAD: 10; 22.5 g (22.0–26.0)
4 (Y-maze – alternation ratio)	Normal	2-way ANOVA	2 month male control: 8; 0.80 (0.65–1.0) 2 month male 5XFAD: 5; 0.70 (0.45–0.75) 2 month female control: 8; 0.73 (0.55–0.90) 2 month female 5XFAD: 8; 0.72 (0.60–1.0) 4 month male control: 7; 0.60 (0.40–0.80) 4 month male 5XFAD: 8; 0.73 (0.60–0.80) 4 month female control: 8; 0.60 (0.45–0.75) 4 month female 5XFAD: 8; 0.65 (0.55–0.80) 8 month male control: 8; 0.65 (0.50–0.90) 8 month male 5XFAD: 7; 0.65 (0.40–0.90) 8 month female control: 9; 0.70 (0.50–0.80) 8 month female 5XFAD: 10; 0.70 (0.55–0.85) 13 month male control: 15; 0.70 (0.55–0.70) 13 month male 5XFAD: 9; 0.50 (0.40–0.60) 13 month female control: 9; 0.70 (0.60–0.85) 13 month female 5XFAD: 10; 0.53 (0.45–0.60)
5 (novel object recognition – proportion of exploration time spent exploring novel object)	Normal	2-way ANOVA	2 month male control: 8; 0.54 (0.48–0.73) 2 month male 5XFAD: 5; 0.58 (0.41–0.70) 2 month female control: 8; 0.60 (0.49–0.75) 2 month female 5XFAD:8; 0.61 (0.36–0.80) 4 month male control: 7; 0.71 (0.41–0.90) 4 month male 5XFAD: 10; 0.56 (0.33–0.71) 4 month female control: 7; 0.67 (0.43–0.76) 4 month female 5XFAD: 6; 0.58 (0.51–0.71) 8 month male control: 9; 0.58 (0.52–0.66) 8 month male 5XFAD:6; 0.55 (0.46–0.72) 8 month female control: 9; 0.67 (0.55–0.79) 8 month female 5XFAD: 10; 0.61 (0.53–0.68) 13 month male control: 15; 0.52 (0.46–0.60) 13 month male 5XFAD: 9; 0.59 (0.39–0.68) 13 month female control: 7; 0.62 (0.50–0.86) 13 month female 5XFAD: 10; 0.54 (0.33–0.67)
6A (open field distance traveled – meters)	Normal	2-way ANOVA	2 month male control: 8; 40.6 (30.6–56.2) 2 month male 5XFAD: 4; 45.7 (33.9–67.5) 2 month female control: 8; 37.6 (21.1–51.2) 2 month female 5XFAD: 8; 50.4 (32.0–89.6) 4 month male control: 7; 37.9 (29.1–58.9) 4 month male 5XFAD: 10; 42.8 (32.6–56.3) 4 month female control: 8; 42.0 (27.0–73.9) 4 month female 5XFAD: 8; 38.9 (18.8–68.3) 8 month male control: 9; 46.8 (35.0–55.0) 8 month male 5XFAD: 7; 53.9 (29.6–87.4) 8 month female control: 9; 49.0 (29.0–64.6) 8 month female 5XFAD: 10; 55.2 (22.9–86.3) 13 month male control: 10; 31.8 (25.0–56.6) 13 month male 5XFAD: 6; 23.9 (17.7–44.0) 13 month female control: 5; 30.6 (22.5–39.5) 13 month female 5XFAD: 7; 37.5 (8.3–59.9)
6B (open field time spent in inner zone – seconds)	Normal	2-way ANOVA	2 month male control: 8; 55.3 (43.3–129.1) 2 month male 5XFAD: 4; 52.4 (17.5–65.6) 2 month female control: 8; 59.9 (22.2–108) 2 month female 5XFAD: 8; 57.6 (13.1–97.0) 4 month male control: 7; 96.1 (44.8–162) 4 month male 5XFAD: 10; 85.4 (38.7–132) 4 month female control: 8; 58.6 (44.2–98.5) 4 month female 5XFAD: 8; 60.8 (15.2–109) 8 month male control: 9; 94.8 (69.4–131) 8 month male 5XFAD: 7; 143.2 (81.4–223) 8 month female control: 9; 80.6 (36.3–116) 8 month female 5XFAD: 10; 90.2 (62.4–132.3) 13 month male control: 13; 105 (71.6–143) 13 month male 5XFAD: 9; 98.8 (67.2–140) 13 month female control: 5; 78.9 (43.2–151) 13 month female 5XFAD: 7; 93.3 (60.9–131)
7A (baseline cold sensitivity – acetone response scores)	Normal	2-way ANOVA	2 month male control: 8; 0.8 (0–1.3) 2 month male 5XFAD: 5; 0.3 (0–0.7) 2 month female control: 8; 1.0 (0.7–1.3) 2 month female 5XFAD: 8; 1.2 (0.3–2.0) 4 month male control: 7; 1.3 (0.7–1.7) 4 month male 5XFAD: 10; 0.7 (0.3–1.3) 4 month female control: 8; 1.0 (0.3–1.3) 4 month female 5XFAD: 8; 1.2 (0.3–1.7) 8 month male control: 9; 0.3 (0.3–0.7) 8 month male 5XFAD: 7; 0.3 (0–1.0) 8 month female control: 9; 0.7 (0.3–1.0) 8 month female 5XFAD: 10; 0.8 (0.3–1.0) 13 month male control: 13; 1.0 (0.3–1.3) 13 month male 5XFAD: 9; 1.0 (0.3–1.0) 13 month female control: 8; 1.0 (0.7–1.7) 13 month female 5XFAD: 10; 1.0 (0.7–1.3)
7B (formalin cold sensitivity – acetone response scores)	Normal	2-way ANOVA	2 month male control: 8; 1.5 (0.7–2.7) 2 month male 5XFAD: 5; 0.7 (0–1.7) 2 month female control: 8; 1.7 (1.3–2.3) 2 month female 5XFAD: 8; 1.3 (1.0–2.0) 4 month male control: 7; 1.0 (0.7–2.3) 4 month male 5XFAD: 10; 1.3 (1.0–1.7) 4 month female control: 8; 1.2 (0.7–3.0) 4 month female 5XFAD: 8; 1.2 (0.7–2.0) 8 month male control: 9; 0.7 (0.3–1.3) 8 month male 5XFAD: 7; 1.3 (0.7–1.3) 8 month female control: 9; 1.0 (1.0–1.7) 8 month female 5XFAD: 10; 1.2 (0.3–1.3) 13 month male control: 13; 2.0 (1.3–2.3) 13 month male 5XFAD: 9; 2.0 (1.3–2.3) 13 month female control: 8; 1.8 (1.0–2.7) 13 month female 5XFAD: 10; 2.3 (1.7–3.0)
8A (baseline mechanical sensitivity – Z scores)	Normal	2-way ANOVA	2 month male control: 4; 0.01(-1.2–1.2) 2 month male 5XFAD: 4; −0.3 (-0.9–1.4) 2 month female control: 4; −0.1 (-1.4–1.5) 2 month female 5XFAD: 4; 0.2 (-1.3–0.9) 4 month male control: 4; 0.4 (-1.5–0.8) 4 month male 5XFAD: 9; −0.1 (-1.1–1.0) 4 month female control: 6; −0.3 (-1.3–1.3) 4 month female 5XFAD: 6; −0.1 (-1.3–1.6) 8 month male control: 9; −0.3 (-0.8–1.7) 8 month male 5XFAD: 5; −0.1 (-1.0–1.5) 8 month female control: 8; −0.1(-1.4–2.1) 8 month female 5XFAD: 5; −0.4 (-0.9–1.7) 13 month male control: 15; −0.2 (-0.7–0.8) 13 month male 5XFAD:8; −0.2 (-1.3–1.1) 13 month female control: 8; −0.3 (-1.0–1.7) 13 month female 5XFAD: 7; −0.5 (-0.6–2.1)
8B (formalin mechanical sensitivity – Z scores)	Normal	2-way ANOVA	2 month male control: 4; 0.2 (-1.3–0.8) 2 month male 5XFAD: 3; −0.1(-0.9–1.0) 2 month female control: 5; 0.1 (-1.4–0.9) 2 month female 5XFAD: 5; 0.2 (-1.4–0.9) 4 month male control: 6; 0.02 (-1.7–1.2) 4 month male 5XFAD: 6; −0.4 (-0.9–1.3) 4 month female control: 4; −0.4 (-0.7–1.5) 4 month female 5XFAD: 7; 0.2 (-1.2–1.3) 8 month male control: 5; 0.1 (-1.5–1.2) 8 month male 5XFAD: 7; −0.4 (-0.9–2.2) 8 month female control: 5; −1.9 (-2.7–0.7) 8 month female 5XFAD: 9; −0.3 (-0.8–0.6) 13 month male control: 15; 0.2 (-0.9–0.4) 13 month male 5XFAD: 8; 0.03 (-1.1–1.6) 13 month female control: 8; −0.4 (-0.7–2.4) 13 month female 5XFAD: 7; −0.5 (-0.7–1.9)
9A (baseline spontaneous pain – grimace scores)	Normal	2-way ANOVA	2 month male control: 8; 0.51 (0.34–0.63) 2 month male 5XFAD: 5; 0.57 (0.16–0.61) 2 month female control: 8; 0.46 (0.42–0.60) 2 month female 5XFAD: 8; 0.47 (0.30–0.67 4 month male control: 7; 0.45 (0.11–0.57) 4 month male 5XFAD: 10; 0.48 (0.27–0.68) 4 month female control: 8; 0.48 (0.35–0.63) 4 month female 5XFAD: 8; 0.50 (0.16–0.71) 8 month male control: 9; 0.57 (0.40–0.68) 8 month male 5XFAD: 5; 0.32 (0.19–0.51) 8 month female control: 9; 0.57 (0.35–0.76) 8 month female 5XFAD: 10; 0.50 (0.38–0.57) 13 month male control: 10; 0.44 (0.25–0.63) 13 month male 5XFAD: 6; 0.40 (0.23–0.88) 13 month female control: 6; 0.38 (0.17–0.63) 13 month female 5XFAD: 7; 0.22 (0.17–0.39)
9B (formalin spontaneous pain – grimace scores)	Normal	2-way ANOVA	2 month male control: 8; 0.47 (0.38–0.74) 2 month male 5XFAD: 5; 0.44 (0.24–0.68) 2 month female control: 8; 0.53 (0.44–0.76) 2 month female 5XFAD: 8; 0.51 (0.27–0.93) 4 month male control: 7; 0.50 (0.42–1.1) 4 month male 5XFAD: 10; 0.56 (0.44–0.81) 4 month female control: 8; 0.57 (0.46–0.82) 4 month female 5XFAD: 8; 0.46 (0.37–0.76) 8 month male control: 9; 0.63 (0.41–0.76) 8 month male 5XFAD: 6; 0.49 (0.23–0.58) 8 month female control: 9; 0.70 (0.56–0.84) 8 month female 5XFAD: 10; 0.64 (0.56–0.70) 13 month male control: 10; 0.72 (0.54–1.0) 13 month male 5XFAD: 6; 0.94 (0.45–1.4) 13 month female control: 6; 0.65 (0.25–1.2) 13 month female 5XFAD: 7; 0.56 (0.38–0.94)
10 (hindlimb clasping – score)	Not Normal	Kruskal-Wallis	2 month male control: 8; 0 (0–2) 2 month male 5XFAD: 5; 0 (0–2) 2 month female control: 8; 0 (0–2) 2 month female 5XFAD: 8; 0 (0–1) 4 month male control: 7; 0 (0–1) 4 month male 5XFAD: 10; 0 (0–1) 4 month female control: 8; 0 (0–2) 4 month female 5XFAD: 8; 0.5 (0–2) 8 month male control: 9; 0 (0–1) 8 month male 5XFAD: 7; 1 (0–3) 8 month female control: 9; 0 (95% C.I. confined to 0) 8 month female 5XFAD: 10; 0.5 (0–2)
11 (formalin response - behaviors per minute)	Normal	2-way ANOVA	13 month male control phase 1: 15; 1.6 (0.8–2.4) 13 month male 5XFAD phase 1: 9; 2.9 (2.0–3.2) 13 month female control phase 1: 9; 1.3 (0.9–2.1) 13 month female 5XFAD phase 1: 10; 1.7 (1.2–2.8) 13 month male control phase 2: 15; 1.4 (1.2–2.2) 13 month male 5XFAD phase 2: 8; 2.3 (1.8–2.8) 13 month female control phase 2: 9; 1.0 (0.5–1.5) 13 month female 5XFAD phase 2: 10; 2.2 (1.4–3.0)

## Results

### Notable amyloid accumulation by 4 months of age

**Fig. 2:**
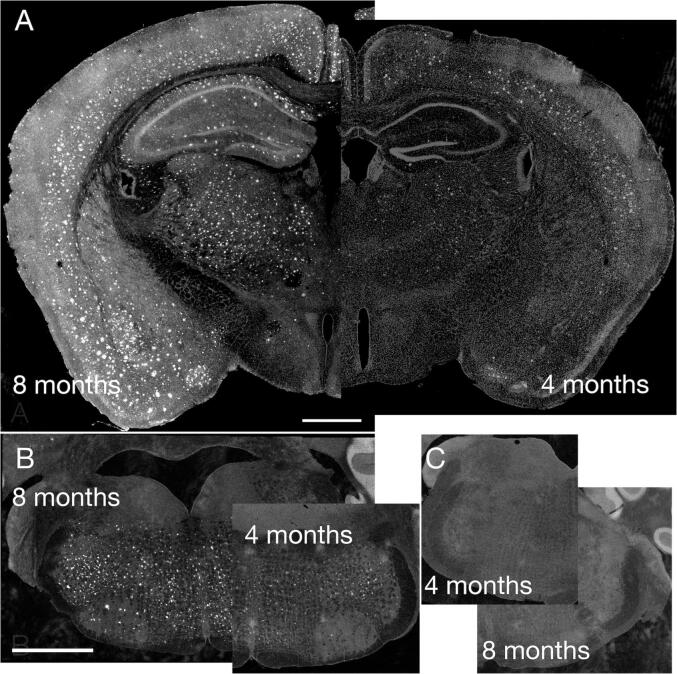
(A) Amyloid throughout the hippocampus and cortex in 4-month and 8-month 5XFAD animals. (B) Amyloid throughout the brainstem, including the RVM, in an 8-month 5XFAD animal. (C) No amyloid detected in the brainstem of 4-month and 8-month control animals. Scale bar = 1 mm.

To confirm that 5XFAD animals displayed expected patterns of amyloid accumulation, we performed an immunofluorescence protocol and qualitatively assessed the presence of amyloid. We found no evidence of amyloid accumulation in control animals at any age. Consistent with previous reports (Oakley, 2006), by 4 months of age amyloid was detected in the cortex and hippocampus of 5XFAD animals (Fig. 2A). We also detected a significant amyloid burden in a descending pain modulating brainstem node that is key in both pain suppression and facilitation: the rostral ventromedial medulla (RVM) (Fig. 2B). Amyloid was absent throughout the brains of age-matched control animals (Fig. 2C). This finding made it plausible that amyloid-induced changes in neural activity within regions such as RVM could alter pain perception and sensory function.

### No body weight deficits at 2 through 8 months of age

**Fig. 3:**
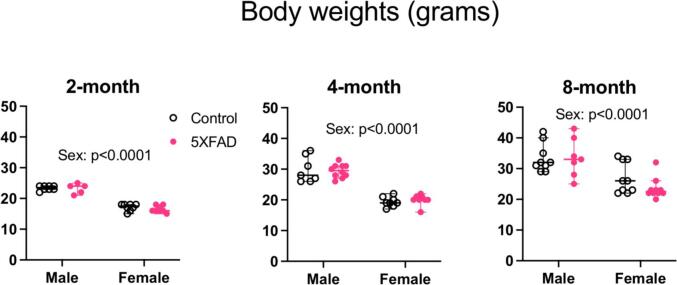
Body weights expressed as grams, at 2, 4 and 8 months of age. There were no differences between control and 5XFAD genotypes at any age. As expected, for each age group, there was a sex difference with males being heavier than females (p < 0.0001). Open data points are from control animals and filled data points are from 5XFAD animals. Each point represents data from one animal. Horizontal bars show the median and error bars show the 95% confidence interval of the median.

We collected body weights of animals at 2, 4 and 8 months of age and performed a 2-way ANOVA, with sex and genotype as variables (Fig. 3: 2 months: n = 8 male controls, n = 5 male 5XFAD, n = 8 female controls, n = 8 female 5XFAD. 4 months: n = 7 male controls, n = 10 male 5XFAD, n = 8 female controls, n = 8 female 5XFAD. 8 months: n = 9 male controls, n = 7 male 5XFAD, n = 9 female controls, n = 10 female 5XFAD). We did not collect body weights at 13 months of age.

As expected, there was an effect of sex on body weight at all ages assessed, with males being heavier than females (2 months: F(1,25) = 231.1, p < 0.0001,Partial η^2^ = 0.90 large effect size, mean male weight = 23.3 g, mean female weight = 16.8 g, difference between means = 6.5 g, 95% C.I. of difference = 5.6–7.4. 4 months: F(1,29) = 123.0, p < 0.0001, Partial η^2^ = 0.81, large effect size, mean male weight = 29.6 g, mean female weight = 19.7 g, difference between means = 9.9 g, 95% C.I. of difference = 8.1–11.8. 8 months: F(1,31) = 25.7, p < 0.0001, Partial η^2^ = 0.45, large effect size, mean male weight = 33.5 g, mean female weight = 25.2 g, difference between means = 8.3, 95% C.I. of difference = 5.0–11.7). There was no effect of genotype on body weight at any age tested (2 months: F(1,25) = 0.88, p = 0.36. 4 months: F(1,29) = 0.009, p = 0.93. 8 months: F(1,31) = 0.99, p = 0.33). There was no interaction between sex and genotype (2 months: F (1, 25) = 0.2791, p = 0.60. 4 months: F (1, 29) = 0.3643, p = 0.55. 8 months: F (1, 31) = 1.149, p = 0.29).

### Assessments of cognitive behavior

#### Spatial memory deficits at 13 months

**Fig. 4:**
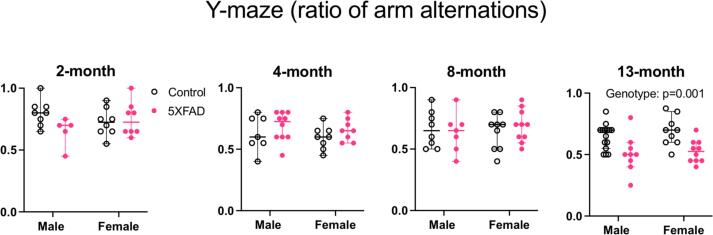
Y-maze performance at 2, 4, 8, and 13 months of age. The ratio of maze arm alternation is represented on each y-axis. There was no difference between control and 5XFAD genotypes at 2, 4 and 8 months. At 13 months, 5XFAD animals had a lower ratio of alternation compared to controls (p = 0.0001). There were no sex differences at any age tested. Open data points are from control animals and filled points are from 5XFAD animals. Each point represents data from one animal. Horizontal bars show the median and error bars show the 95% confidence interval of the median.

We used the Y-maze task to assess spatial memory in 5XFAD mice relative to their control counterparts at 2, 4, 8 and 13 months of age. For data collected at each age, we ran a 2-way ANOVA with sex and genotype as variables (Fig. 4: 2 months: n = 8 male controls, n = 8 female controls, n = 5 male 5XFAD, n = 8 female 5XFAD. 4 months: n = 7 male controls, n = 8 female controls, n = 8 male 5XFAD, n = 8 female 5XFAD. 8 months: n = 8 male controls, n = 9 female controls, n = 7 male 5XFAD, n = 10 female 5XFAD. 13 months: n = 15 male controls, n = 9 female controls, n = 9 male 5XFAD, n = 10 female 5XFAD).

There was no difference in y-maze performance based on sex (2 months: F(1,25) = 0.077, p = 0.78. 4 months: F(1,29) = 0.64, p = 0.43. 8 months: F(1,30) = 0.095, p = 0.76. 13 months: F(1,39) = 0.80, p = 0.38). Only at 13 months of age did 5XFAD animals show smaller maze arm alternation ratios (22% reduction) compared to controls (2 months: F(1,25) = 1.91, p = 0.18. 4 months: F(1,29) = 2.26, p = 0.14. 8 months: F(1,30) = 0.06, p = 0.81. 13 months: F(1,39) = 18.33, p = 0.0001, Partial η^2^ = 0.32, large effect size, mean 13-month control = 0.67, mean 13-month 5XFAD = 0.52, difference between means = 0.15, 95% C.I. of difference = 0.08–0.23). There was no interaction between sex and genotype (2 months: F (1, 25) = 3.751, p = 0.06. 4 months: F (1, 29) = 0.005879, p = 0.94. 8 months: F (1, 30) = 0.6128, p = 0.44. 13 months: F (1, 39) = 0.1264, p = 0.72).

#### Intact short-term object recognition through 13 months of age

**Fig. 5:**
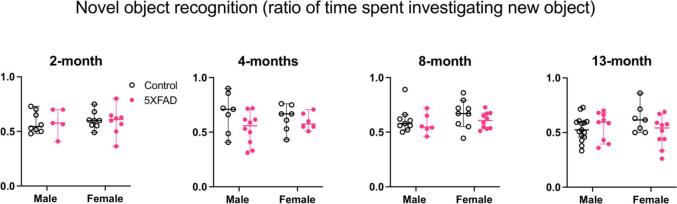
Novel object recognition performance at 2, 4, 8, and 13 months of age. The ratio of time spent investigating the new object relative to the familiar object is represented on each y-axis. There were no sex differences and no differences between control and 5XFAD genotypes at any age tested. Open data points are from control animals and filled points are from 5XFAD animals. Each point represents data from one animal. Horizontal bars show the median and error bars show the 95% confidence interval of the median.

We used the novel object recognition task to measure short-term memory. For data collected at each age, we ran a 2-way ANOVA with sex and genotype as variables (Fig. 5: 2 months: n = 8 male controls, n = 5 male 5XFAD, n = 8 female controls, n = 8 female 5XFAD. 4 months: n = 7 male controls, n = 10 male 5XFAD, n = 7 female controls, n = 6 female 5XFAD. 8 months: n = 9 male controls, n = 6 male 5XFAD, n = 9 female controls, n = 10 female 5XFAD. 13 months: n = 15 male controls, n = 9 male 5XFAD, n = 7 female controls, n = 10 female 5XFAD).

There was no effect of sex at any age tested (2 months: F(1,25) = 0.068, p = 0.80. 4 months: F(1,26) = 0.029, p = 0.87. 8 months: F(1,30) = 1.38, p = 0.25. 13 months: F(1,37) = 0.22, p = 0.64). There was no difference between genotypes in the amount of time spent exploring the new object at any age (2 months: F(1,25) = 0.008, p = 0.93. 4 months: F(1,26) = 2.96, p = 0.097. 8 months: F(1,30) = 1.67, p = 0.21. 13 months: F(1,37) = 1.24, p = 0.27). There was no interaction between sex and genotype (2 months: F (1, 25) = 0.06718, p = 0.80. 4 months: F (1, 26) = 1.164, p = 0.29. 8 months: F (1, 30) = 0.1143, p = 0.74. 13 months: F (1, 37) = 2.837, p = 0.10). These data suggest that these 5XFAD mice experience no loss of short-term object memory compared to age-matched controls.

#### Transient sex-specific changes in anxiety-associated behavior

To test general locomotion and anxiety-associated behaviors we used the open field test. In this test, mice prefer to spend time in the outer zone of the field, and more time spent in the outer region relative to the inner region is thought to indicate a more anxious phenotype (Prut and Belzung, 2003).

**Fig. 6:**
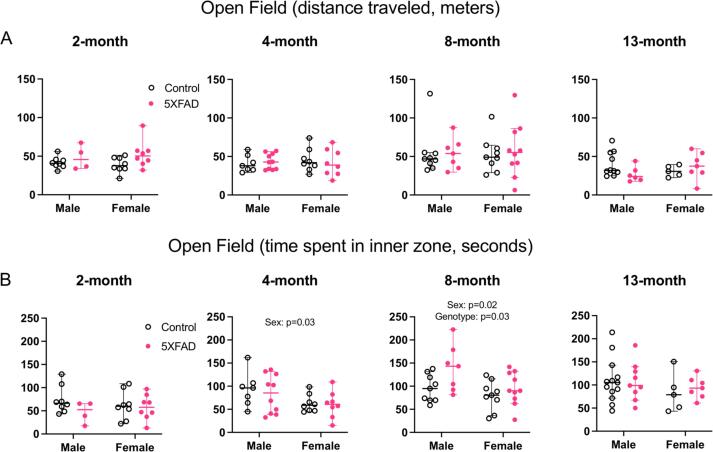
Open field behavior in 2, 4, 8 and 13-month animals. (A) Total distance traveled (meters) is represented on each y-axis. There were no sex differences and no genotype differences at any age tested. (B) Time spent in the inner zone (seconds) is represented on each y-axis. At 4 and 8 months of age, males spent more time in the inner zone relative to females (4 months: p = 0.03, 8 months: p = 0.02). At 8 months, 5XFAD animals spent more time in the inner zone compared to controls (p = 0.03). There was no interaction between sex and genotype at 8 months. Open data points are from control animals and filled points are from 5XFAD animals. Each point represents data from one animal. Horizontal bars show the median and error bars show the 95% confidence interval of the median.

For each age group, we ran a 2-way ANOVA with sex and genotype as variables (Fig. 6: 2 months: n = 8 male controls, n = 4 male 5XFAD, n = 8 female controls, n = 8 female 5XFAD. 4 months: n = 7 male controls, n = 10 male 5XFAD, n = 8 female controls, n = 8 female 5XFAD. 8 months: n = 9 male controls, n = 7 male 5XFAD, n = 9 female controls, n = 10 female 5XFAD. 13 months: n = 13 control males for inner zone time and n = 10 for distance traveled, n = 9 male 5XFAD for inner zone time and n = 6 for distance traveled, n = 5 female controls, n = 7 female 5XFAD).

To measure general locomotion, we collected the total distance traveled by each mouse, in meters, throughout the 10-minute open field test. We ran a 2-way ANOVA with sex and genotype as variables, finding no effect of sex at any age (2 months: F(1,24) = 0.03, p = 0.87. 4 months: F(1,29) = 0.08, p = 0.77. 8 months: F(1,31) = 0.02, p = 0.90. 13 months: F(1,24) = 0.03, p = 0.87). There was also no effect of genotype on total distance traveled in any of the age groups (2 months: F(1,24) = 4.01, p = 0.06. 4 months: F(1,29) = 0.007, p = 0.93. 8 months: F(1,31) = 0.05, p = 0.82. 13 months: F(1,24) = 0.37, p = 0.55). There was no interaction between sex and genotype (2 months: F (1, 24) = 0.5084, p = 0.48. 4 months: F (1, 29) = 0.4145, p = 0.52. 8 months: F (1, 31) = 0.08797, p = 0.77. 13 months: F (1, 24) = 3.327, p = 0.08). This suggests that general locomotor activity is the same in 5XFAD animals compared to age-matched control animals.

More time spent in the inner zone of the open field suggests that animals are less anxious. At 4 months of age, but not at 2 months, there was an effect of sex on time spent in the inner zone, with 4-month males spending 42% more time there compared to 4-month females (2 months: F(1,24) = 0.002, p = 0.97 4 months: F(1,29) = 5.08, p = 0.03, Partial η^2^ = 0.15, medium effect size, mean male = 88.0 sec, mean female = 61.6 sec, difference between means = 26.4 sec, 95% C.I. of difference = 2.4–50.3). At these ages, genotype had no effect on time spent in the inner zone (2 months: F(1,24) = 1.94, p = 0.18. 4 months: F(1,29) = 0.28, p = 0.60). At 8 months of age, there was a main effect of genotype and a main effect of sex on time spent in the inner zone of the field, with males spending more time there than females, and 5XFAD spending more time in the inner zone than controls (Genotype: F(1,31) = 5.48, p = 0.026, Partial η^2^ = 0.15, medium effect size, control mean = 87.5 sec, 5XFAD mean = 116.8 sec, difference between means = 29.2 sec, 95% C.I. of difference = 3.8–54.7 sec. Sex: F(1,31) = 5.76, p = 0.023, Partial η^2^ = 0.16, medium effect size, male mean = 117.1 sec, female mean = 87.2 sec, difference between means = 30.0 sec, 95% C.I. of difference = 4.5–55.4 sec). At 13 months of age, there was no effect of sex (F(1,30) = 1.66, p = 0.21) or of genotype (F(1,30) = 0.081, p = 0.78) on time spent in the inner zone. At all ages, there was no interaction between sex and genotype (2 months: F (1, 24) = 0.9917, p = 0.33. 4 months: F (1, 29) = 0.09228, p = 0.76. 8 months: F (1, 31) = 1.310, p = 0.26. 13 months: F (1, 30) = 0.1709, p = 0.68).

Taken together, these data suggest that at 8 months of age, there is transiently reduced anxiety in male mice compared to females, and in 5XFAD animals compared to controls.

### Assessments of sensory profiles

#### No differences in baseline cold sensitivity

**Fig. 7:**
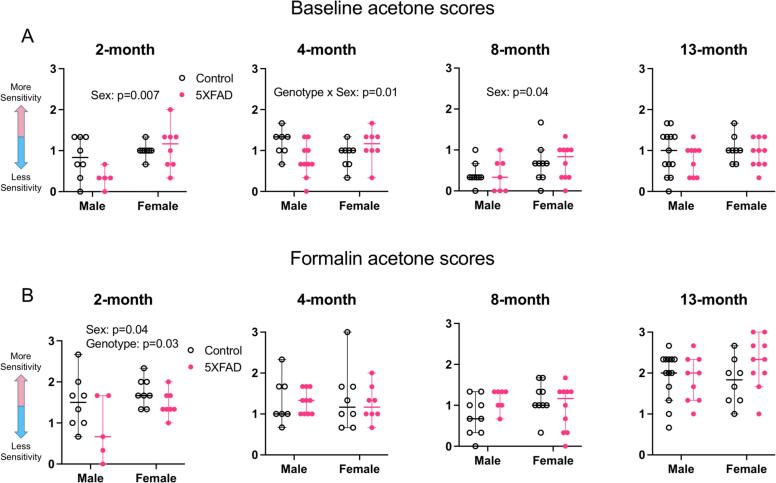
Cold sensitivity. All y-axes depict acetone response scores. (A) Responses to acetone at baseline. At 2 months and 8 months, females had higher response scores than males (2 months: p = 0.007, 8 months: p = 0.04). At 4 months, there was no main effect of sex or genotype, but there was an interaction between sex and genotype (p = 0.01). (B) Responses to acetone after hindpaw formalin injection. At 2 months, females had higher response scores than males (sex: p = 0.04) and 5XFAD animals had lower response scores than controls (genotype: p = 0.03). Open data points are from control animals and filled points are from 5XFAD animals. Each point represents data from one animal. Horizontal bars show the median and error bars show the 95% confidence interval of the median.

To test for cold sensitivity, we compared acetone response scores in control and 5XFAD animals at 2, 4, 8, and 13 months of age. For each age group, we performed a 2-way ANOVA with sex and genotype as variables (Fig. 7A: 2 months: n = 8 male controls, n = 5 male 5XFAD, n = 8 female controls, n = 8 female 5XFAD. 4 months: n = 7 male controls, n = 10 male 5XFAD, n = 8 female controls, n = 8 female 5XFAD. 8 months: n = 9 male controls, n = 7 male 5XFAD, n = 9 female controls, n = 10 female 5XFAD. 13 months: n = 13 control males, n = 9 male 5XFAD, n = 8 female controls, n = 10 female 5XFAD).

At 2 months, females had higher acetone response scores compared to males (F(1,25) = 8.77, p = 0.007, Partial η^2^ = 0.26, large effect size, mean male score = 0.58, mean female score = 1.04, difference between means = 0.46, 95% C.I. of difference = 0.14–0.78). There was no effect of genotype on acetone scores at 2 months (F(1,25) = 1.81, p = 0.19). There was no interaction between sex and genotype at 2 months (F (1, 25) = 3.551, p = 0.07).

At 4 months, there was an interaction between sex and genotype (F(1,29) = 6.76, p = 0.01, Partial η^2^ = 0.19, medium effect size), but no main effect of sex (F(1,29) = 0.03, p = 0.87) and no main effect of genotype (F(1,29) = 0.45, p = 0.51).

At 8 months, there was a main effect of sex, with females having higher scores compared to males (F(1,31) = 4.42, p = 0.04, Partial η^2^ = 0.12, medium effect size, mean male score = 0.39, mean female score = 0.68, difference between means = 0.29, 95% C.I. of difference = 0.009–0.57). There was no effect of genotype on acetone response scores at 8 months (F(1,31) = 0.0007, p = 0.98). There was no interaction between sex and genotype at 8 months (F (1, 31) = 0.04024, p = 0.84).

At 13 months, there was no effect of sex (F(1,36) = 0.83, p = 0.37) and no effect of genotype (F(1,36) = 1.05, p = 0.31) on acetone response scores. There was also no interaction between sex and genotype at 13 months (F (1, 36) = 0.05286, p = 0.82).

These data suggest that, while sex differences manifest at some ages, there is no difference in cold sensitivity in 5XFAD animals compared to controls at any age tested.

#### Less cold sensitivity during formalin challenge at 2 months

We next compared cold sensitivity in the context of persistent inflammatory pain by injecting formalin into the hindpaw and performing the acetone response test. For each age group, we performed a 2-way ANOVA with sex and genotype as variables (Fig. 7B: 2 months: n = 8 male controls, n = 5 male 5XFAD, n = 8 female controls, n = 8 female 5XFAD. 4 months: n = 7 male controls, n = 10 male 5XFAD, n = 8 female controls, n = 8 female 5XFAD. 8 months: n = 9 male controls, n = 7 male 5XFAD, n = 9 female controls, n = 10 female 5XFAD. 13 months: n = 13 control males, n = 9 male 5XFAD, n = 8 female controls, n = 10 female 5XFAD).

At 2 months, there was a main effect of sex, with females having higher scores than males (F(1,25) = 4.58, p = 0.04, Partial η^2^ = 0.15, medium effect size, mean male score = 1.18, mean female score = 1.60, difference between means = 0.42, 95% C.I. of difference = 0.02–0.83). There was also a main effect of genotype at 2 months, with 5XFAD animals having lower response scores than controls (F(1,25) = 5.53, p = 0.03, Partial η^2^ = 0.18, medium effect size, mean control score = 1.63, mean 5XFAD score = 1.16, difference between means = 0.46, 95% C.I. of difference = 0.06–0.87). There was no interaction between sex and genotype (F(1.25) = 0.75, p = 0.39). This suggests that 5XFAD mice have less cold sensitivity in the context of formalin pain at 2 months of age.

At 4, 8, and 13 months, there was no effect of sex (4 months: F(1,29) = 0.0006, p = 0.98. 8 months: F(1,31) = 0.41, p = 0.53. 13 months: F(1,36) = 0.56, p = 0.46) or of genotype (4 months: F(1,29) = 0.18, p = 0.67. 8 months: F(1,31) = 0.36, p = 0.56. 13 months: F(1,36) = 1.14, p = 0.29) on acetone response scores. There was no interaction between sex and genotype at these ages (4 months: F (1, 29) = 0.05947, p = 0.81. 8 months: F (1, 31) = 3.981, p = 0.05. 13 months: F (1, 36) = 2.090, p = 0.16).

#### Baseline tactile sensitivity is unchanged

**Fig. 8:**
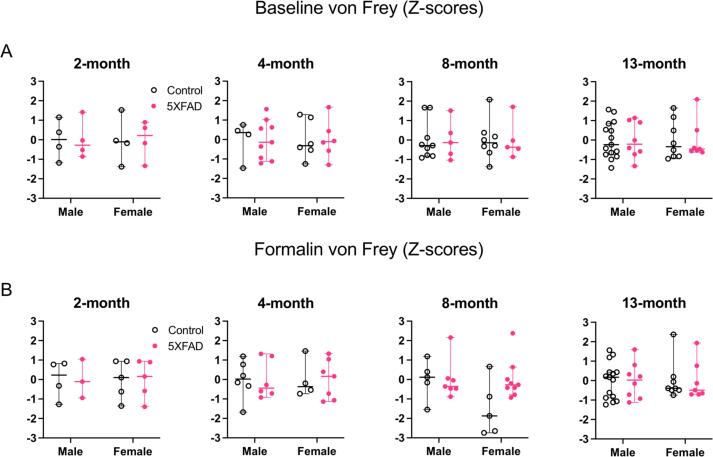
Mechanical sensitivity. All y-axes depict von Frey thresholds represented as Z-scores. (A) Responses to hindpaw mechanical stimulation at baseline. There were no differences based on sex or genotype at any age tested. (B) Responses to mechanical stimulation after hindpaw formalin injection. There were no differences based on sex or genotype at any age tested. Open data points are from control animals and filled points are from 5XFAD animals. Each point represents data from one animal. Horizontal bars show the median and error bars show the 95% confidence interval of the median.

To assess tactile sensitivity, we performed the hindpaw von Frey test of mechanical thresholds at 2, 4, 8 and 13 months of age. Due to inter-experimenter variability, we transformed each animal’s threshold to a z-score. We performed a 2-way ANOVA with sex and genotype as variables for each age group (Fig. 8A: 2 months: n = 4 male controls, n = 4 male 5XFAD, n = 4 female controls, n = 4 female 5XFAD. 4 months: n = 4 male controls, n = 9 male 5XFAD, n = 6 female controls, n = 6 female 5XFAD. 8 months: n = 9 male controls, n = 5 male 5XFAD, n = female controls, n = 5 female 5XFAD. 13 months: n = 15 male controls, n = 8 male 5XFAD, n = 8 female controls, n = 7 female 5XFAD).

At all ages, there was no effect of sex on mechanical sensitivity (2 months: F(1,12) = 0.0003, p = 0.99. 4 months: F(1,21) = 7 × 10^−15^, p > 0.99. 8 months: F(1,23) = 6.2 × 10^−16^, p > 0.99. 13 months: F(1,34) = 4.94 × 10^−17^, p > 0.99). There was no effect of genotype at any of the ages tested (2 months: F(1,12) = 0.0003, p > 0.99. 4 months: F(1,21) = 2.7 × 10^−15^, p > 0.99. 8 months: F(1,23) = 4.9 × 10^−16^, p > 0.99. 13 months: F(1,34) = 2.21 × 10^−15^, p > 0.99). There was no interaction between sex and genotype (2 months: F (1, 12) = 0.0003, p = 0.99. 4 months: F (1, 21) = 1.4 × 10^−16^ p > 0.99. 8 months: F (1, 23) = 3.0 × 10^−20^, p > 0.99. 13 months: F (1, 34) = 5.0 × 10^−15^, p > 0.99). These data suggest that 5XFAD animals have tactile sensitivity that is comparable to their age-matched counterparts.

##### No differences in tactile sensitivity during a formalin challenge

To determine whether tactile sensitivity in the context of inflammatory pain differs between genotypes, we performed hindpaw von Frey testing after injecting the hindpaw with formalin. We again transformed mechanical thresholds to z-scores, due to inter-experimenter variability. We performed a 2-way ANOVA with sex and genotype as variables for each age group (Fig. 8B: 2 months: n = 4 male controls, n = 3 male 5XFAD, n = 5 female controls, n = 5 female 5XFAD. 4 months: n = 6 male controls, n = 6 male 5XFAD, n = 4 female controls, n = 7 female 5XFAD. 8 months: n = 5 male controls, n = 7 male 5XFAD, n = 5 female controls, n = 9 female 5XFAD. 13 months: n = 15 male controls, n = 8 male 5XFAD, n = 8 female controls, n = 7 female 5XFAD).

At 2, 4, 8 and 13 months there was no effect of sex on tactile sensitivity (2 months: F(1,13) = 3.1 × 10^−17^, p > 0.99. 4 months: F(1,19) = 7.1 × 10^−15^, p > 0.99. 8 months: F(1,22) = 2.9, p = 0.10. 13 months: F(1,34) = 6.3 × 10^−18^, p > 0.99). At all ages tested, there was also no effect of genotype on tactile sensitivity (2 months: F(1,13) = 1.5 × 10^−15^, p > 0.99. 4 months: F(1,19) = 3.4 × 10^−15^, p > 0.99. 8 months: F(1,22) = 2.9, p = 0.10. 13 months: F(1,34) = 3.5 × 10^−15^, p > 0.99). There was no interaction between sex and genotype at any age tested (2 months: F (1, 13) = 8.9 × 10^−15^, p > 0.99. 4 months: F (1, 19) = 3.4 × 10^−15^, p > 0.99. 8 months: F (1, 22) = 2.9, p = 0.10. 13 months: F (1, 34) = 1.3 × 10^−15^, p > 0.99. This suggests that tactile sensitivity during formalin inflammation is not altered in 5XFAD animals compared to controls.

#### 8-month old 5XFAD animals show fewer signs of ongoing pain at baseline

**Fig. 9:**
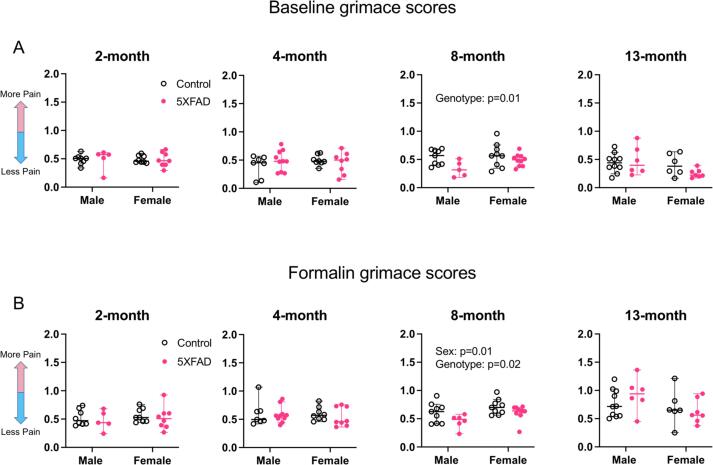
Ongoing pain assessed by facial grimace. All y-axes depict grimace scores. (A) Grimace scores at baseline. At 8 months, 5XFAD animals had lower grimace scores compared to controls (p = 0.01). (B) Grimace scores after hindpaw formalin injection. At 8 months, females had higher grimace scores than males (p = 0.01) and 5XFAD animals had lower grimace scores than controls (p = 0.02). There was no interaction between sex and genotype. Open data points are from control animals and filled points are from 5XFAD animals. Each point represents data from one animal. Horizontal bars show the median and error bars show the 95% confidence interval of the median.

To determine whether 5XFAD animals experience more spontaneous, ongoing pain compared to controls, we performed grimace scoring in animals at 2, 4, 8, and 13 months of age. Grimace scoring involves quantifying changes in specific facial expressions such as orbital tightening, ear position, and nose or cheek position in order to generate a grimace score for a given animal. In this paradigm, the lowest score of 0 indicates that the animal is showing no signs of pain and the highest score of 2 suggests that the animal is experiencing extreme spontaneous pain. For each age group, we performed a 2-way ANOVA with sex and genotype as variables (Fig. 9A: 2 months: n = 8 male controls, n = 5 male 5XFAD, n = 8 female controls, n = 8 female 5XFAD. 4 months: n = 7 male controls, n = 10 male 5XFAD, n = 8 female controls, n = 8 female 5XFAD. 8 months: n = 9 male controls, n = 5 male 5XFAD, n = 9 female controls, n = 10 female 5XFAD. 13 months: n = 10 male controls, n = 6 male 5XFAD, n = 6 female controls, n = 7 female 5XFAD).

There was no effect of sex on baseline grimace scores at any age tested (2 months: F(1,25) = 0.0001, p = 0.99. 4 months: F(1,29) = 0.28, p = 0.60. 8 months: F(1,29) = 2.99, p = 0.09. 13 months: F(1,25) = 3.64, p = 0.07).

There was no effect of genotype on baseline grimace scores at 2, 4, and 13 months (2 months: F(1,25) = 0.0005, p = 0.98. 4 months: F(1,29) = 0.16, p = 0.69. 13 months: F(1,25) = 0.74, p = 0.40). At 8 months, there was an effect of genotype on baseline grimace score, with 5XFAD animals having 26% lower scores than controls (F(1,29) = 6.93, p = 0.01, Partial η^2^ = 0.19, medium effect size, mean control score = 0.56, mean 5XFAD score = 0.41, difference between means = 0.15, 95% C.I. of difference = 0.03–0.26). There was no interaction between sex and genotype at any age tested (2 months: F (1, 25) = 0.006, p = 0.94. 4 months: F (1, 29) = 1.46, p = 0.24. 8 months: F (1, 29) = 1.24, p = 0.27. 13 months: F (1, 25) = 2.05 , p = 0.16). This suggests that at 8 months, 5XFAD animals have less ongoing pain relative to age-matched controls.

#### 8-month old 5XFAD animals show fewer signs of ongoing formalin-evoked pain

To determine whether 5XFAD animals experience more spontaneous, ongoing pain compared to controls when challenged with inflammation, we performed grimace scoring in animals after hindpaw formalin injection at 2, 4, 8, and 13 months of age. To confirm that the formalin was effective as intended, we compared baseline and formalin grimace scores in control animals. At all ages but 2 months, formalin grimace scores were higher than baseline scores (2 month baseline 0.49, 2 month formalin 0.52, p = 0.42; 4 month baseline 0.48, 4 month formalin 0.56, p = 0.02; 8 month baseline median 0.57, 8 month formalin median 0.66, p = 0.02; 13 month baseline 0.29, 13 month formalin 0.68, p = 0.0003). To assess genotype and age-based differences, for each age group, we performed a 2-way ANOVA with sex and genotype as variables (Fig. 9B: 2 months: n = 8 male controls, n = 5 male 5XFAD, n = 8 female controls, n = 8 female 5XFAD. 4 months: n = 7 male controls, n = 10 male 5XFAD, n = 8 female controls, n = 8 female 5XFAD. 8 months: n = 9 male controls, n = 6 male 5XFAD, n = 9 female controls, n = 10 female 5XFAD. 13 months: n = 10 male controls, n = 6 male 5XFAD, n = 6 female controls, n = 7 female 5XFAD).

There was no effect of sex at 2, 4, and 13 months (2 months: F(1,25) = 0.49, p = 0.49. 4 months: F(1,29) = 0.16, p = 0.69. 13 months: F(1,25) = 3.4, p = 0.08). There was also no effect of genotype at 2, 4, and 13 months (2 months: F(1,25) = 0.62, p = 0.44. 4 months: F(1,29) = 0.41, p = 0.53. 13 months: F(1,25) = 0.18, p = 0.68).

At 8 months, there was a main effect of sex, with females having higher grimace scores than males (F(1,30) = 6.92, p = 0.01, Partial η^2^ = 0.19, medium effect size, mean score females = 0.66, mean score males = 0.53, difference between means = 0.13, 95% C.I. of difference = 0.03–0.23). At 8 months there was also a main effect of genotype, with 5XFAD animals having lower grimace scores compared to controls (F(1,30) = 6.33, p = 0.02, Partial η^2^ = 0.17, medium effect size, mean score controls = 0.66, mean score 5XFAD = 0.53, difference between means = 0.13, 95% C.I. of difference = 0.02–0.23). At all ages tested, there was no interaction between sex and genotype (2 months: F (1, 25) = 0.001, p = 0.97. 4 months: F (1, 29) = 0.113, p = 0.74. 8 months: F (1, 30) = 0.277, p = 0.60. 13 months: F (1, 25) = 1.34, p = 0.26). This suggests that when challenged with formalin, 8-month-old 5XFAD animals experience somewhat less ongoing pain compared to controls.

#### No sensorimotor pathology in 5XFAD animals

**Fig. 10:**
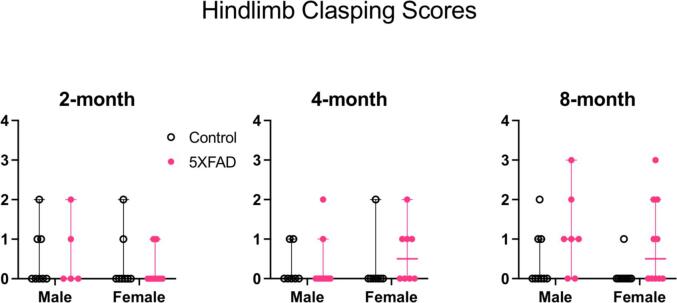
Hindlimb clasping scores at 2, 4, and 8, months of age. The clasping score is represented on each y-axis. There was no difference between control and 5XFAD genotypes at 2 or 4 months. At 8 months, 5XFAD animals had a higher clasping score compared to controls (p = 0.02). There were no sex differences at any age tested. Open data points are from control animals and filled points are from 5XFAD animals. Each point represents data from one animal. Horizontal bars show the median and error bars show the 95% confidence interval of the median.

To assess the progression of sensorimotor deficits, we scored hindlimb clasping behavior at 2, 4 and 8 months of age. We did not complete this test in animals at 13 months of age, due to the lack of clear sensory abnormalities in 5XFAD animals. Because the data were not normally distributed, we performed a Kruskal-Wallis test for each age group (Fig. 10: 2 months: n = 8 male controls, n = 5 male 5XFAD, n = 8 female controls, n = 8 female 5XFAD. 4 months: n = 7 male controls, n = 10 male 5XFAD, n = 8 female controls, n = 8 female 5XFAD. 8 months: n = 9 male controls, n = 7 male 5XFAD, n = 9 female controls, n = 10 female 5XFAD).

There was no difference between groups at 2 months (Kruskal-Wallis statistic = 0.75, p = 0.86), 4 months (Kruskal-Wallis statistic = 2.6, p = 0.46), or 8 months (Kruskal-Wallis statistic = 6.8, p = 0.08). This finding suggests that by 8 months of age, 5XFAD animals do not show signs of sensorimotor pathology as manifested by abnormal hindlimb clasping behavior.

#### Increased formalin-induced behaviors at 13 months

**Fig. 11:**
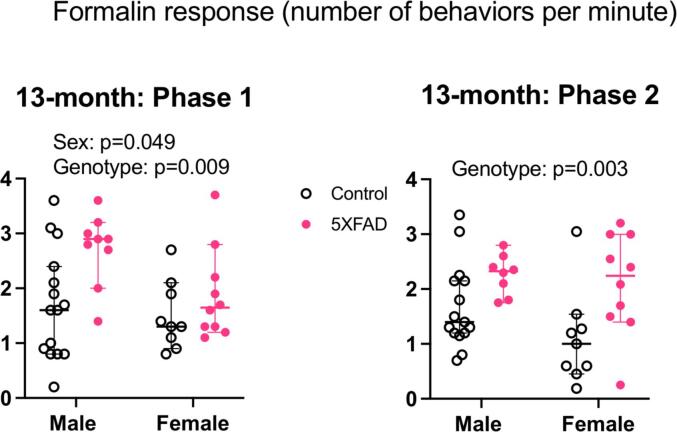
Behavioral responses to formalin in 13-month old animals. Each y axis shows the number of formalin-induced behaviors per minute, with the maximum possible score being 4. Data were collected in the 30 min following hindpaw formalin injection. Phase 1 encompasses the first 10 min after injection and phase 2 is from 10 to 30 min post-injection. In phase 1, males showed more formalin-induced behaviors per minute compared to females (p = 0.049) and 5XFAD animals exhibited more behaviors compared to controls (p = 0.009). There was no interaction between sex and genotype. In phase 2, 5XFAD animals showed more formalin-induced behaviors compared to controls (p = 0.003). Open data points are from control animals and filled points are from 5XFAD animals. Each point represents data from one animal. Horizontal bars show the median and error bars show the 95% confidence interval of the median.

The reflexive pain tests performed during formalin testing did not yield a clear pattern of functionally significant differences in pain responses. To confirm that formalin did induce the expected behavioral reactions, we quantified spontaneous pain-related behaviors after hind paw formalin injection in a subset of 13 month old animals. We filmed mice for 30 min after injecting formalin into the hindpaw. Because formalin-induced inflammatory pain occurs in two phases, we scored behaviors separately for the first 10 min (phase 1) and the last 20 min (phase 2). For each phase, we performed a 2-way ANOVA on the number of formalin-induced pain behaviors per minute, with sex and genotype as variables (Fig. 11: n = 15 male controls, n = 9 male 5XFAD for phase 1 and 8 male 5XFAD for phase 2, n = 9 female controls, n = 10 female 5XFAD).

In phase 1, there was a main effect of sex, with males showing more pain behaviors per minute compared to females (F(1,39) = 4.13, p = 0.049, Partial η^2^ = 0.10, medium effect size, mean behaviors males = 2.2, mean behaviors females = 1.7, difference between means = 0.52, 95% C.I. of difference = 0.002–1.0). There was also a main effect of genotype in phase 1, with 5XFAD animals showing more pain behaviors per minute than controls (F(1,39) = 7.5, p = 0.009, Partial η^2^ = 0.16, medium effect size, mean behaviors controls = 1.6, mean behaviors 5XFAD = 2.3, difference between means = 0.70, 95% C.I. of difference = 0.18–1.2). There was no interaction between sex and genotype (F(1,39) = 1.6p = 0.22)

In phase 2, there was no effect of sex (F(1,38) = 2.3, p = 0.14). There was a main effect of genotype, with 5XFAD animals showing more pain behaviors per minute compared to controls (F(1,38) = 10.5, p = 0.003, Partial η^2^ = 0.22, medium effect size, mean control behaviors = 1.4, mean 5XFAD behaviors = 2.2, difference between means = 0.8, 95% C.I. of difference = 0.30–1.3). There was no interaction between sex and genotype (F(1,38) = 0.78, p = 0.38).

These findings indicate that at 13 months of age, 5XFAD animals display more pain behaviors compared to age-matched controls when challenged with persistent inflammatory pain. However, this difference had only a medium statistical effect size, and a small physiological effect size.

## Discussion

### 5XFAD animals have significant early amyloid accumulation

5XFAD mice displayed amyloid in the cortex and hippocampus by 4 months of age, consistent with earlier reports (Oakley, 2006, Liu et al., 2017). We also found a heavy burden of amyloid in the rostral ventromedial medulla (RVM), a key brain region in descending pain modulation and sensory processing (Heinricher et al., 2009). Early RVM amyloid accumulation in 5XFAD animals has not been previously reported, to our knowledge.

### Amyloid accumulations are expected to generate significant cellular consequences

Amyloid exposure alone can lead to increased neuronal excitability (Busche, 2012, Busche et al., 2008). Amyloid presence can induce both glial activation and neuronal cell death (Klyubin, 2008, Mattson, 1997, Smale et al., 1995, Kobayashi et al., 2002, Kobayashi et al., 2005, Bayer and Wirths, 2011). Indeed, neuronal loss has been shown in 5XFAD animals (Oakley, 2006, Ali et al., 2019). Thus, it is plausible that amyloid-induced changes throughout the brain, including in pain-processing regions, shape neural activity and functional outcomes. This finding supported our endeavor to evaluate sensory behaviors in 5XFAD animals and to determine whether pain sensitivity is altered in correlation with amyloid accumulation.

*5XFAD animals exhibit minimal differences in pain responses.* The prevalence of both pain and Alzheimer’s disease increase with age (Duncan et al., 2011). Pain prevalence is high in patients with dementia, and this pain is under-diagnosed and under-treated (Li et al., 2015, van Kooten et al., 2015, T. Binnekade et al., 2017). Here, we examine early changes in somatosensory profiles and pain responses in a mouse model of amyloid accumulation – an area of study with sparse existing data, compared to cognitive function assessments. We found no compelling differences in sensory function or pain metrics in 5XFAD animals. The only difference was in male mice, which showed less cold sensitivity at early ages and fewer signs of ongoing pain at 8 months, with modest effect sizes.

We used formalin injections to model persistent inflammatory pain. In mice, formalin injection consistently evokes pain in two distinct phases. The first phase waxes within 5 min after injection and involves direct activation of nociceptors. The second phase is usually recorded between 15 and 30 min post-formalin in mice (Hunskaar et al., 1985, Rosland, 1991) and involves activity of inflammatory mediators and spinal sensitization. While the peak of phase 2 occurs in this 15–30 min post-injection time frame, elevated formalin-induced behaviors can persist through 60 min (Abbott et al., 1995). Thus, we captured both phase 1 and 2 responses during our 30-minute window for formalin scoring and grimace assessment. We note, however, that our subsequent mechanical and cold sensitivity testing lasted beyond the peak phase 2 response.

To confirm that our formalin injections were effective, we quantified formalin-evoked behaviors in a subset of 13-month-old animals. We counted licking, lifting and guarding, which are commonly assessed formalin-induced pain behaviors (Abbott et al., 1995, Coderre and Melzack, 1992, Saddi and Abbott, 2000). We opted for a non-weighted scoring method, in which each behavior was given an equivalent score. Evidence suggests that quantifying multiple behaviors improves scoring correlations with formalin concentration, and that weighted and non-weighted scoring paradigm options are comparable (Saddi and Abbott, 2000).

Thirteen-month-old 5XFAD animals showed more pain behaviors than age-matched controls, but the effect size was not large, and is unlikely to have functional significance. Coupled with this finding, 5XFAD animals at any age, including 13 months, did not show increased grimace scores when challenged with formalin. This suggests that 5XFAD animals do not experience more spontaneous, formalin-evoked pain. While our data do not support a compelling difference in formalin pain responses, we recognize that studies implementing models of chronic pain or neuropathic pain might yield different results. We also acknowledge that different mouse strains may have different pain sensitivity and response behaviors. Both C57BL/6J and SJL/J strains contribute to the background strain for the animals used in this study, with potentially varying proportions in each generation. In models of inflammation, these strains have different response profiles to mechanical stimuli (Lu et al., 2012). This variability may contribute to variations in sensory responses, thereby masking the effects of the 5XFAD mutations.

Overall, our findings suggest that amyloid burden and its downstream consequences in 5XFAD mice are insufficient to induce meaningful changes in pain and sensory processing.

### Amyloid burden in 5XFAD mice does not translate into early cognitive decline

Downstream from cellular disruption, amyloid accumulations can have significant functional correlations. Amyloid is considered a hallmark of Alzheimer’s Disease (Reitz and Mayeux, 2014). Despite the heavy amyloid burden in the 5XFAD mice studied here, we did not observe the anticipated early-onset cognitive deficits. Modest deficits in Y-maze spatial memory emerged between 8 and 13 months; at all ages tested, we detected no deficits in short-term novel object recognition.

### Y-maze

Other studies of 5XFAD mice report variable cognitive deficits. When assessing spatial memory using the Y-maze test, some found that 5XFAD deficits emerge as early as 4–5 months of age (Oakley, 2006). Others report that moderate deficits emerge at 5 months (Hayashi et al., 2019), 6 months, (Shukla et al., 2013, Devi and Ohno, 2012), 7 months (Wei et al., 2016), or 8 months (de Pins, 2019). In the latter study, 5XFAD animals perform with maze arm alternations close to chance level (50%), similar to the performance we report at 13 months (de Pins, 2019). In another study, 5XFAD animals show alternation ratios above 60% at 6 months, and just under 60% at 13 months (Sosna et al., 2018).

### Novel object recognition

Similarly, 5XFAD deficits in short-term (minutes to hours) and long-term (≥1 day) object memory are variable. Some studies report short-term memory deficits in 4-month-old 5XFAD mice (Giannoni et al., 2013). Others show that, in 4 month-olds, novel object recognition impairments only manifest when the interval between familiarization and recall reaches 4 h; when this interval is 10 min, 1 h, or 24 h, there are no differences between 5XFAD and wild-type mice (Kim et al., 2020). At older ages (6–8 months), some groups report impaired long-term object memory in 5XFAD animals (Tohda et al., 2012). In contrast, another study finds that at 12 months, but not at 6 months of age, 5XFAD mice display impaired long-term object recognition (Maiti et al., 2021). Some studies report no object memory deficits: one group, using a 1-minute inter-trial interval, found no novel object recognition deficits in 8.5-month old 5XFAD animals (Braun and Feinstein, 2019). Using a 2-hour inter-trial interval, another study reports that 8–10 month old 5XFAD and wild-type animals perform comparably (Kubota et al., 2016).

### Open field and sensorimotor pathology

5XFAD animals may show decreased anxiety-like behavior in open field or elevated plus maze assays (Schneider et al., 2014, Jawhar et al., 2012). Supporting our findings, another group found that 8-month old male 5XFAD mice have decreased anxiety-like behavior (Braun and Feinstein, 2019). We found that 5XFAD animals displayed decreased anxiety-associated behavior compared to controls at 8 months, however, this difference was no longer apparent at 13 months of age. This might reflect an overall reduced activity level with aging, in both control and 5XFAD animals. We found no locomotor deficits in 13-month-old 5XFAD animals compared to age-matched controls. Furthermore, at younger ages, sensorimotor pathology, as assessed by hindlimb clasping, was absent. It is possible that a lack of spinal pathology could underlie this comparable performance, in contrast to the coupling of spinal pathology and motor dysfunction that others observed in these animals (Jawhar et al., 2012, Li et al., 2013, Xie et al., 2019).

Variations in the onset and severity of a behavioral phenotype is not specific to 5XFAD animals. Other models of amyloid accumulation also yield variable patterns and timing of cognitive deficits (Kosel et al., 2020, Webster et al., 2013) (reviewed by Webster et al., 2014). The literature, in conjunction with our findings, suggest that, despite consistent early amyloid burden, 5XFAD mice have notable variation in the timing and degree of cognitive decline. Thus, amyloid burden might not predict the time course and severity of cognitive decline in mice. Indeed, in humans there is no strong correlation between amyloid burden and cognitive decline (Khosravi, 2019, Nelson et al., 2012).

### Sensory function in other mouse models of Alzheimer’s Disease

Studies formally characterizing sensory function in different Alzheimer’s disease mouse models are not abundant (Reviewed by Lawn et al., 2021). In our hands, sensory evaluations did not yield a distinct functional difference between 5XFAD and wild-type animals. In other models of amyloid accumulation, such as the TASTPM mouse strain carrying two amyloid-related mutations, there is evidence of altered sensory function. For example, TASTPM mice at 4 months showed thermal sensitivity profiles comparable to wild-type animals, but at 8 months a pattern of reduced thermal nociceptive thresholds emerged in the transgenic animals (Aman et al., 2016). Similarly, TASTPM mice from 6 to 8 months of age have a reduced sensitivity to joint inflammation elicited in a model of osteoarthritis (Aman et al., 2019). In contrast, 3xTg mice, despite displaying both amyloid and neurofibrillary tangle pathology, show no differences in baseline thermal sensory thresholds (Filali et al., 2012, Cañete and Giménez-Llort, 2021). Thus, genetic differences and experimental design are crucial in determining whether transgenic mice appropriately model the pattern of sensory changes seen clinically.

*Future directions may lie beyond amyloid.* Amyloid plaque accumulation is a pathological hallmark of Alzheimer’s disease. Early-onset and inherited forms of the disease involve amyloid production and processing protein mutations. Individuals with Trisomy 21 (the amyloid precursor protein gene is on this chromosome) have an increased risk of developing early-onset Alzheimer’s disease (Lott and Head, 2019). Amyloid accumulation, however, is only one neuropathological hallmark of Alzheimer’s Disease – the others include hyperphosphorylated tau accumulation and neuronal loss. Thus, while 5XFAD animals may model important pathological findings of Alzheimer’s disease, they do not fully recapitulate this clinical condition.

Progressive cognitive decline is a distinctive feature of Alzheimer’s disease, however, 5XFAD mice do not consistently model this process with a reproducible timeline. Throughout the literature, reports on the timing and severity of decline in tasks requiring spatial memory and object memory are notably variable. Even when cognitive differences are clear between 5XFAD and control groups, effect sizes are moderate. As outlined above, in studies of cognitive function in 5XFAD animals, experimental context can significantly shape outcomes. We acknowledge that there are many methodological nuances that might account for different outcomes in behavioral assays, both in our experiments and across the literature.

Despite behavioral variability, 5XFAD mice undoubtedly provide a robust and reliable model of amyloid accumulation, making them useful in disentangling the relationships between amyloid and surrounding cells. Although the functional outcomes associated with these pathological changes are varied, models such as 5XFAD will continue to prove crucial in elucidating the complex cellular and molecular interactions stemming from amyloid accumulation.

## Funding Sources

This work was supported by NIH grants to AK (grant numbers R01NS099245 and R01NS069568).

## Author contributions

OU: data curation, formal analysis, investigation, writing – original draft, writing – review and editing. KA: investigation, formal analysis, writing – review and editing. CR: investigation, writing – review and editing. BG: investigation, writing – review and editing. AK: conceptualization, data curation, formal analysis, funding acquisition, writing – original draft, writing – review and editing.

## Declaration of Conflict of Interest

The authors declare that they have no known conflict of interest regarding the publication of this paper.
